# A Rare Case of Immune Reconstitution Inflammatory Syndrome Development in an Immunocompromised Patient with Progressive Multifocal Leukoencephalopathy and Multicentric Castleman's Disease

**DOI:** 10.1155/2013/460701

**Published:** 2013-08-19

**Authors:** Mohankumar Kurukumbi, Sheldon Steiner, Shariff Dunlap, Sherita Chapman, Noha Solieman, Annapurni Jayam-Trouth

**Affiliations:** ^1^Department of Neurology, Howard University Hospital, 2041 Georgia Avenue, Washington, DC 20060, USA; ^2^Department of Neurology, Howard University College of Medicine, Washington, DC 20060, USA; ^3^Department of Neurology, University of Virginia, Charlottesville, VA 22908, USA

## Abstract

Immune reconstitution inflammatory syndrome (IRIS) development in HIV with preexistent progressive multifocal leukoencephalopathy (PML) has been extensively studied. PML-IRIS typically manifests clinically as new or worsening neurologic symptoms in conjunction with enlarging CNS lesions and occurs in approximately 10–20 percent of HIV-infected patients with PML who begin HAART. Likewise, Multicentric Castleman's Disease (MCD), a rare malignant lymphoproliferative disorder, has a strong and well-known association with HIV. Our case provides a rare instance of PML-IRIS in combination with MCD in an HIV-positive individual. The combination of all three diseases has never been reported in the literature. Both MCD and PML were present during initial determination of HIV infection in our patient and their disease courses were altered during the subsequent development of IRIS.

## 1. Introduction

PML is a severe demyelinating disease of the CNS caused by reactivation of the human polyomavirus JC. The vast majority of PML cases occur in HIV-infected patients with varying clinical manifestations. IRIS is a paradoxical inflammatory response that occurs following the initiation of HAART in HIV-infected patients. IRIS development in conjunction with PML typically manifests clinically as new or worsening neurologic symptoms in conjunction with enlarging CNS lesions. MCD is a rare primary disease of the lymph nodes and presents in older individuals with constitutional symptoms such as fever, night sweats, weight loss, and weakness or fatigue [1, 2]. It has a strong association with HIV.

Our case presentation outlines a distinctive coexistent presentation of IRIS, PML, and MCD in a HIV positive patient. The occurrence of all three pathologies together has not been previously documented in the literature. 

## 2. Case Presentation

The patient is a 46-year-old African American female with a past medical history of hypertension and noncompliance with medication, who presented with progressive dysarthria over a 10-day period that had worsened in the previous 3-4 days. The patient denied any other neurological symptoms. On neurological examination, the patient was found to have difficulties with speech and decreased vision due to preexisting glaucoma. The rest of the neurological exam was normal. The patient was admitted for the evaluation of subacute progressive motor dysphasia.

**Figure 1:**
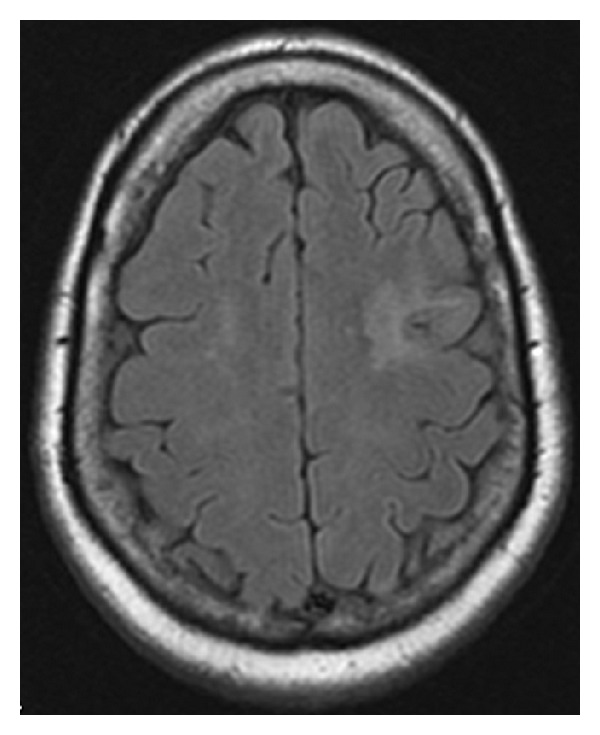
MRI Brain-axial T2 FLAIR showing left frontal lesion.

During the hospital stay, an MRI of the brain was performed for the suspicion of stroke or demyelination etiology. The MRI brain with and without contrast showed a left frontal lesion, which was not typical for ischemic infarction or demyelination (Figure 1). This subsequently broadened the differential to include sarcoidosis, PML, CMV encephalitis, and CNS lymphoma.

There was also significant cervical lymphadenopathy seen on MRI as well as axillary lymphadenopathy found on the physical examination. HIV testing was performed for further evaluation in view of new differentials, and the patient was found to be HIV positive with CD4 count of 26.

**Figure 2:**
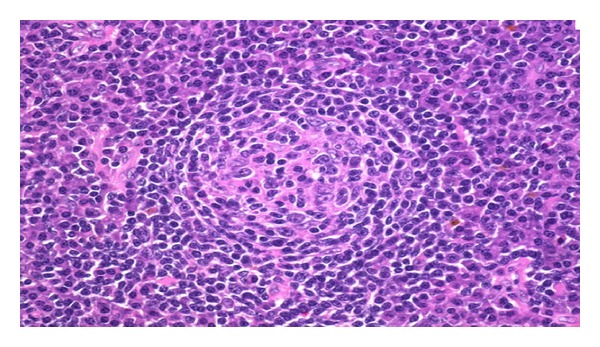
Axillary lymph node biopsy showing plasma cell proliferation in the interfollicular area.

Both lumbar puncture and axillary biopsy were also performed. The results of the lumbar puncture were positive for JC virus and negative for CMV, HSV-1, HSV-2, and EBV viruses. The diagnosis of PML was made. Biopsy of the axillary nodes revealed atypical lymphoid hyperplasia with regressed germinal centers, paracortical expansion, and increased plasma cells, which suggested a diagnosis of MCD (Figure 2). The patient experienced new onset seizures during the hospitalization as well. After ruling out other potential causes, it was determined that the patient had a focal seizure most likely secondary to her brain lesion. The patient was started on HAART and eventually discharged on antiepileptic medications and *Pneumocystis jirovecii* prophylaxis. 

**Figure 3:**
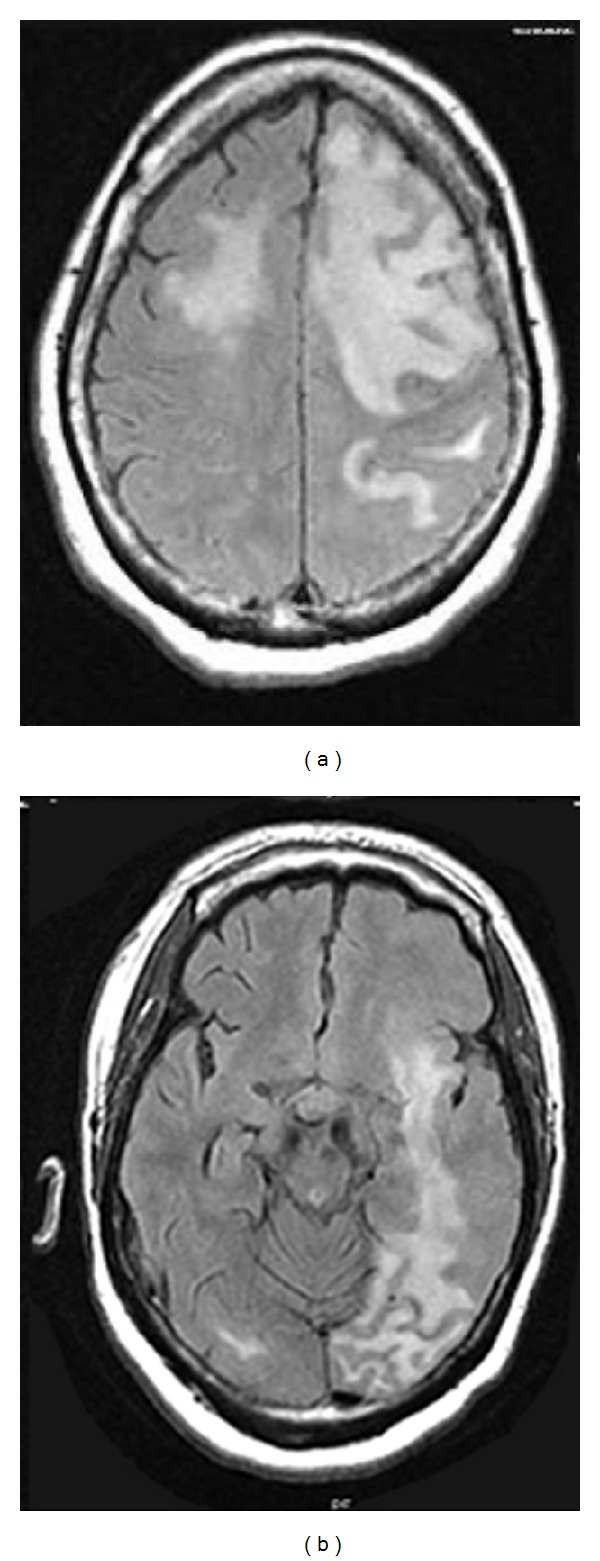
MRI brain-diffuse increased FLAIR signal throughout the left cerebral hemisphere.

About three months later, the patient presented with complaints of new-onset right-sided weakness. The CD4 count had increased diminutively from 26 to 29 during these three months. The patient was assessed to have possible immune reconstitution inflammatory syndrome (IRIS) given the new-onset right-sided weakness. MRI with and without contrast showed diffuse bilateral cerebral hemispheric edema, worst on the left cerebral hemisphere which crossed the corpus callosum anteriorly into the right frontal lobe and posteriorly into the left parietooccipital lobe (Figures 3(a) and 3(b)). JC virus was detected in the patient's serum as well as in the CSF again via lumbar puncture. The patient was continued on HAART for PML and additionally placed on dexamethasone after considering IRIS. The patient declined to have a biopsy of the brain lesion. She had a hospital course that was further complicated by worsening kidney and liver functions as well as a urinary tract infection. She was treated for these conditions and subsequently sent to an intensive care nursing home in stable condition.

## 3. Discussion

PML is a severe demyelinating disease of the CNS caused by reactivation of the human polyomavirus JC. The vast majority of PML cases occur in immunocompromised patients, particularly HIV-infected patients. The prevalence of PML increased substantially during the HIV epidemic with approximately 5% of patients with AIDS developing the disease [3]. PML is predominately caused by infection of oligodendrocytes. Therefore, the resultant clinical neurological deficits are associated with areas of demyelination in the brain. More recent studies have established that JC virus also causes infection of cortical neurons and that demyelinating lesions of PML can frequently involve gray matter [4]. Therefore, the clinical presentation can vary to include altered mental status, motor deficits (hemiparesis or monoparesis), sensory deficits, hemianopsia, cognitive dysfunction, aphasia, and/or coordination and gait difficulties. Typically, the optic nerves and spinal cord are spared. Seizures can take place in up to 18 percent of patients with PML, usually occurring when the white matter lesions are located immediately adjacent to the cortical grey matter [5]. Most patients with coexistent HIV and PML are severely immunosuppressed with CD4 counts <200 per mm^3^. Our patient's measured CD4 count was 26 which put her at risk for subsequent PML development. 

Radiographic findings of PML typically show symmetric or asymmetric multifocal white matter lesions without edema, mass effect, or contrast enhancement [6]. On CT scan, the lesions appear as hypodense or patchy areas and can be easily overlooked. Typical MRI findings include hyperintense areas on T2-weighted and FLAIR and hypointense areas on T1-weighted images [7]. Although lesions can be present in grey matter structures that contain myelinated fibers (e.g., basal ganglia or thalamus), they are most commonly found in subcortical hemispheric white matter or cerebellar peduncles. The MRI findings of our patient showed a left temporal lesion, consistent with PML. The usual means of making a diagnosis of PML is via detection of viral DNA or proteins by in situ hybridization, immunohistochemistry on a brain biopsy sample, or detection of JC virus DNA in the CSF by PCR [7]. The latter of these three options, detection of JC virus DNA in the CSF, was performed in our case. 

Immune reconstitution inflammatory syndrome (IRIS) is a paradoxical inflammatory response to clinically apparent or subclinical pathogens following the initiation of HAART in HIV-infected patients. By definition, IRIS involves an inflammatory component occurring in the setting of immune reconstitution that cannot be explained by drug toxicity, a new opportunistic infection (OI), or the expected course of a previously diagnosed OI. The clinical features of IRIS are thought to result from exaggerated and dysregulated cellular immune responses and are linked to the location and type of preexisting infections. Preexisting infections are wideranging and include mycobacterial and cryptococcal infections, cytomegalovirus, *Pneumocystis jirovecii*, Kaposi's sarcoma, non-Hodgkin lymphoma, and PML. Risk factors for the development of IRIS in HIV-infected individuals include antiretroviral naiveté, using a boosted protease inhibitor, low CD4 lymphocyte counts (<100 cells/mm^3^), higher level of viremia at baseline, rapid decrease in HIV load, rapid immune recovery following the initiation of HAART, and the presence of active or subclinical opportunistic infections at the time of initiation of HAART [8, 9]. Our patient's antiretroviral naiveté, CD4 count <50, and active opportunistic infection at the time of initiation of HAART were risk factors for the development of IRIS. A 2010 systematic review and meta-analysis [10] determined the overall incidence of IRIS in patients starting HAART to be approximately 13%. Higher incidence rates have been reported when patients with known OIs are studied [9]. A separate 2010 prospective trial found that the incidence of IRIS occurred at a mean of 33 days following the initiation of HAART [11], with most cases developing within the first 3 months. Our patient had a known OI (JC virus) discovered soon after initiating HAART and developed clinical symptoms and MRI findings indicative of IRIS approximately three months after initial presentation. 

IRIS development due to a preexistent JC virus infection leads to the onset of PML or worsening of previously diagnosed PML. PML-IRIS typically manifests clinically as new or worsening neurologic symptoms in conjunction with enlarging CNS lesions. It occurs in approximately 10–20 percent of HIV-infected patients with PML who begin HAART [12, 13]. The enlarging CNS lesions often show contrast enhancement (56.7% of cases) which is usually absent in classic PML lesions [14]. Contrast enhancement is thought to be due to development of an inflammatory response with breakdown of the blood-brain barrier (altered permeability and integrity of brain vessels) [14]. The inflammation can subsequently lead to brain edema, swelling, and/or mass effect which are also not typically seen in classic PML lesions. Biopsies performed in a few cases of PML-IRIS have shown demyelination typical of PML and surrounding inflammation containing large amounts of CD8+ lymphocytes [7]. Cinque et al. [13] determined that most cases of IRIS associated with JC virus infections occurred within three to six weeks after HAART initiation. It was also determined that patients with preexisting PML developed IRIS earlier, had larger lesion loads on brain MRI, and had a higher mortality rate in comparison to patients who developed PML and IRIS simultaneously [14]. Three months after administration of HAART to our patient, she developed new-onset right-sided weakness and rapidly progressing MRI findings indicating IRIS development with preexistent PML. MRI was repeated from her initial visit and showed PML progression too rapid to be explained by the expected course of classic PML. Diffuse bilateral cerebral hemispheric edema, which crossed the corpus callosum, was a new MRI finding and is not typically seen in classic PML lesions. Despite the minimal increase in CD4 count in our patient following administration of HAART, these described clinical and MRI findings point toward IRIS development. Brain biopsy was not performed in our patient due to refusal of consent. 

In patients with PML-IRIS, the standard treatment is continuation of HAART therapy in addition to high-dose glucocorticoid therapy (e.g., dexamethasone). However, the efficacy and safety of using steroids in PML-IRIS remain largely unknown due to limited data. A combination of neurologic deterioration and radiologic evidence of brain swelling is a relative indication for steroid use [7, 14]. In three separate case reports, HAART was discontinued for 2-3 weeks following diagnosis of PML-IRIS, and the patients did well on the followup. However, the practice of discontinuing HAART is not currently advocated [15]. Following the diagnosis of PML-IRIS in our patient, HAART was continued, and dexamethasone was added in an attempt to counteract the cerebral edema with midline shift. This corresponds to the standard treatment of PML-IRIS. 

CD is a rare primary disease of the lymph nodes. It consists of two distinct disease groups with significantly different prognoses: unicentric CD (UCD) and multicentric CD (MCD). UCD (or localized CD) typically involves a solitary asymptomatic lymphoproliferative disorder of young adults and is not associated with human herpesvirus 8 (HHV-8). In contrast MCD presents in older individuals (median age of 60) with constitutional symptoms such as fever, night sweats, weight loss, and weakness or fatigue [1, 2]. Peripheral lymphadenopathy, splenomegaly, and laboratory abnormalities such as anemia, hypoalbuminemia, and elevated sedimentation rate are characteristically present. MCD also differs from UCD in having a strong association with HIV and HHV-8 infection [1, 2].

There are three major variants of CD: hyaline vascular variant, plasma cell variant, and HHV8+ CD. Our patient's axillary node biopsies showed atypical lymphoid hyperplasia with regressed (or atrophic) germinal centers, paracortical expansion, and increased plasma cells, consistent with the plasma cell variant of CD [16]. 

CD is most often seen in HIV-positive individuals as MCD, rather than UCD. HHV8 has been found to be a nearly universal factor in HIV-associated MCD. For instance, HHV8 DNA sequences were detected in 14 of 14 cases of HIV-positive MCD in one study [1, 17]. As mentioned previously, an immunohistochemical stain with HHV8 was negative in our patient, but it is likely that PCR amplification would have determined the presence of HHV8 DNA sequences following DNA extraction.

Risk factors for the development of MCD in HIV-positive patients include nadir CD4 count >200/microL, increased age, no previous HAART exposure, and non-Caucasian ethnicity [18]. Risk factors present in our patient include no previous HAART exposure and non-Caucasian ethnicity. Disease progression is variable, although HIV-infected patients tend to have a relatively acute course (median duration of three months of symptoms at the time of diagnosis) [2]. Initiation of HAART in patients who are positive for both HIV and MCD may increase the likelihood of developing fulminant MCD, but this is as yet unconfirmed and was not definitively shown in our case [19].

## 4. Conclusion

Our case provides a rare instance of PML-IRIS in combination with MCD in an HIV-positive individual. The combination of all three diseases has never been reported in the literature. Lymph node enlargement in end-stage HIV/AIDS usually raises the suspicion of lymphoma or HIV lymphadenopathy, rather than CD. Both CD and PML were present during initial determination of HIV infection in our patient, and their disease courses were altered during the subsequent development of IRIS. PML-IRIS in particular was shown via evolving MRI and clinical findings. 

## Abbreviations

IRIS: Immune reconstitution inflammatory syndrome
PML: Progressive multifocal leukoencephalopathy
MCD: Multicentric Castleman's disease
HIV: Human immunodeficiency virus
AIDS: Acquired immunodeficiency syndrome
MRI: Magnetic resonance imaging
CMV: Cytomegalovirus
CNS: Central nervous system
CD4: Cluster of differentiation 4
HSV-1: Herpes simplex virus-1
HSV-2: Herpes simplex virus-2
EBV: Epstein-Barr virus
HAART: Highly active antiretroviral therapy
CSF: Cerebrospinal fluid
FLAIR: Fluid attenuated inversion recovery
DNA: Deoxyribonucleic acid
PCR: Polymerase chain reaction
OI: Opportunistic infection
UCD: Unicentric Castleman's disease.
